# Ferritin as a diagnostic, differential diagnostic, and prognostic marker for immune-related adverse events

**DOI:** 10.20892/j.issn.2095-3941.2021.0037

**Published:** 2021-08-11

**Authors:** Weihong Zhang, Yuan Meng, Lin Yang, Meng Shen, Li Zhou, Runmei Li, Yang Wang, Weijiao Du, Yanjuan Xiong, Ying Han, Xinwei Zhang, Liang Liu, Xiubao Ren

**Affiliations:** 1 Department of Immunology and Biotherapy, Key Laboratory of Cancer Immunology and Biotherapy, Tianjin Medical University Cancer Institute and Hospital, National Clinical Research Center for Cancer, Key Laboratory of Cancer Prevention and Therapy, Tianjin, Tianjin’s Clinical Research Center for Cancer, Tianjin 300060, China; 2 State Key Laboratory of Experimental Hematology, Peking Union Medical College, Institute of Hematology and Blood Diseases Hospital, Chinese Academy of Medical Sciences, Tianjin 300020, China

**Keywords:** Ferritin, diagnosis, prognosis, irAEs, PD-1, chemotherapy

## Abstract

**Objective:**

Distinguishing immune-related adverse events (irAEs) caused by immune checkpoint inhibitors (ICIs) from the AEs caused by chemotherapy, targeted therapy, or infection is highly difficult. This study offers new insights into evaluating the diagnosis, differential diagnostic, and prognostic value of ferritin for irAEs induced by ICIs.

**Methods:**

From December 1, 2018, to April 1, 2019, we examined 318 patients with malignant tumors who received serum ferritin monitoring. The cohort comprised 231 patients treated with PD-1 inhibitor or combination with chemotherapy, and 87 patients treated with chemotherapy. Of the 231 patients, 90 had irAEs (irAE group), 70 had non-irAEs (non-irAE group), 67 had no AEs (no irAE-non irAE group), and 4 had unclassified AEs. In the 87 patients, 60 had AEs (AE group), and 27 had no AEs (no AE group). Statistical analyses were conducted with nonparametric Mann-Whitney tests.

**Results:**

At the onset of AEs in the irAE group, ferritin (normal range, 35–150 µg/L) rose to a median of 927 µg/L (range, 117–17,825 µg/L) from 86 µg/L at baseline (range, 29–421 µg/L) (*P* < 0.001). Ferritin levels at the onset of AEs in the irAE group were significantly higher than those in the non-irAE group (median, 81 µg/L; range, 32–478 µg/L) (*P* < 0.001) and the AE group (median, 103 µg/L; range, 23–712 µg/L) (*P* < 0.001). After treatment in the irAE group, ferritin continuously decreased to a normal range in recovered patients, showed no significant changes in stable patients, and continued to rise in patients who died.

**Conclusions:**

Ferritin can be used as a diagnostic, differential diagnostic, and prognostic marker for irAEs in patients treated with ICIs.

## Introduction

Immune checkpoint inhibitors (ICIs) targeting cytotoxic T-lymphocyte-associated antigen 4 (CTLA-4) or programmed cell death 1 receptor (PD-1)/programmedcell death 1 ligand 1 (PD-L1) pathways are influencing new cancer treatment model sand producing unprecedented clinical effects in a wide diversity of cancers, such as melanoma, renal cell carcinoma, lung cancer, urothelial cancer, or head and neck cancer^1–6^. However, these treatments induce a series of distinct immune-related adverse events (irAEs), which are similar to autoimmune responses. Virtually every organ of the body is affected by irAEs, such as pneumonitis, colitis, endocrinopathies, and hepatitis; rarer adverse effects include myocarditis and neurotoxicity^7,8^. The gastrointestinal, pulmonary, dermatologic, hepatic, and endocrine systems are thereby frequently affected. The ASCO Clinical Practice Guideline Summary provides system-based toxicity diagnosis and management guidelines, which recommend treatments depending on the affected system. The major treatment for ICI-related AEs is immunosuppression with methylprednisolone^8^. Because of the adverse effects of steroid-refractory, immunosuppressive effects are in facilitating escalation, including other immunosuppressants like tocilizumab, mycophenolate mofetil, or infliximab^8^. Although these irAE symptoms are usually reversible and controllable, they often lead to treatment disruption or dose reduction, and/or discontinuation of checkpoint treatment. When new symptoms appear during ICI treatment, other etiological factors such as infections, viral reactivation, tumor progression, or toxicity associated with other drugs must be excluded. Because a host of organ system symptoms are unspecific in irAE, diagnosis and differential diagnosis have become difficult. Especially with a combination of immunotherapy with chemotherapy or targeted therapy, distinguishing irAEs from AEs caused by chemotherapy or targeted therapy is very difficult. The diagnosis and differential diagnosis of irAEs play a major role in the treatment of AE and in subsequent tumor treatment decisions.

Recently, Abolhassani et al.^8^ have evaluated the value of CRP in diagnosing irAEs through retrospective analysis of the correlations among 88 events of irAEs in 37 patients with melanoma and their serumc-reactive protein (CRP) levels. Elevated CRP was predictive of irAEs in patients without infectious disease who were treated with ICIs. Liudahl and colleagues^9^ have reported a relationship between enhanced risk of high-grade irAEs in patients treated with ICIs and inchoate therapy-induced changes in circulating B cells. Bamias^10^ has shown that levels of CRP pre-therapy, combined with IFN-γ and IL-17, may be used to predict irAE development. These study designs were retrospective; therefore, a control group without AEs or with non-irAEs was not included. ICIs combined with chemotherapy or other therapies are increasingly and widely used in tumor treatment, and distinguishing irAEs from the AEs caused by chemotherapy or other treatments will be difficult. Thus, markers for convenient and rapid detection and differentiation of irAEs from infections or other AEs are urgently needed. At present, no effective marker can distinguish irAEs from infections or other AEs. Serum ferritin, a marker of inflammation, infection, and malignancy, continues to be measured in patients with cancer. On October 28, 2018, a patient with adrenal carcinoma and PD-L1 > 50% in tumor tissues received PD-1 inhibitor treatment in our department. After 1 day of treatment, the patient had panic symptoms. Electrocardiography indicated bigeminy with premature ventricular contraction. Myocardial enzyme indexes were abnormally elevated. Serum ferritin increased from 67 µg/L at baseline (normal range, 35–150 µg/L) to 7,680 µg/L. After 3 days, the patient developed heart failure, renal failure, and respiratory failure. The patient was diagnosed with the grade 4 irAE of explosive autoimmune myocarditis and received high-dose methylprednisolone, mycophenolate mofetil, high-dose immunoglobulin, and plasma exchange to treat the irAE. After 30 days of treatment, the patient recovered from the irAE. During the 30 days of treatment for the irAE, ferritin and myocardial enzymes simultaneously decreased to normal levels. Therefore, we wondered whether ferritin might serve as a diagnostic and prognostic indicator of irAE. Consequently, we designed this study to evaluate the diagnostic, differential diagnostic, and prognostic value of ferritin in irAEs induced by ICIs.

## Materials and methods

### Patients

This study was approved by the Ethical Committee of Tianjin Medical University Cancer Institute and Hospital, according to the guidelines of the Declaration of Helsinki (Approval No. E2016055). Informed consent was obtained from all participants. Patients who were eligible for enrollment were required to meet the following criteria: a diagnosis of solid tumor or acute leukemia; age between 18 and 75 years; treatment with anti-PD-1 antibody (nivolumab, pembrolizumab, sintilimab, or camrelizumab), anti-PD-1 antibody combination with chemotherapy or chemotherapy; and sufficient blood samples to test for serum ferritin levels. Patients were excluded if they had immune deficiency or autoimmune diseases, severe allergic disorder, uncontrollable medical conditions, or infectious diseases such as viral reactivation, colitis, or pneumonia.

### AE diagnosis, treatment, and ferritin monitoring

All patients received hemato-biochemistry and imaging examinations at baseline according to the National Cancer Institute Common Terminology Criteria for Adverse Events (CTCAE) version 4.0 and the National Comprehensive Cancer Network Management of Immunotherapy-Related Toxicties version 1, 2018. The changes in hemato-biochemistry and imaging examination were monitored during the entire adverse reaction. Adverse events (AEs) and abnormal laboratory findings were graded on the basis of CTCAE version 4.0 and the National Comprehensive Cancer Network Management of Immunotherapy-Related Toxicties version 1, 2018. If patients who received ICI or an ICI combination with chemotherapy had AEs, the AEs were further diagnosed as irAEs or non-irAEs by a multi-disciplinary team including an oncologist, rheumatologist, immunologist, radiologist, and pathologist. IrAEs were managed by the team throughout the entire treatment process. All patients received serum ferritin monitoring every 3 days, including 3 consecutive tests for patients with no AEs and continuous detection until AE recovery, or up to 10 tests in patients with irAEs or non-irAEs. Patients with irAEs received treatment according to the National Comprehensive Cancer Network Management of Immunotherapy-Related Toxicties version 1, 2018.

### Statistical analysis

Statistical differences between uninterrupted ferritin values were calculated with nonparametric Mann-Whitney tests. A 2-sided *P* value less than 0.05 indicated statistical significance. All calculations were implemented in R software version 3.5.1.

## Results

### Patient characteristics

From December 1, 2018, to April 1, 2019, 318 patients who met the eligibility criteria received serum ferritin monitoring, including 231 patients treated with PD-1 inhibitor or a combination with chemotherapy, and 87 patients treated with chemotherapy (**Table 1**). Among the 231 patients, 90 had irAEs (irAE group), 70 had non-irAEs (non-irAE group), 67 had no AEs (no irAE-non-irAE group), and 4 had unclassified AEs. Among the 87 patients, 60 had AEs (AE group), and 27 had no AEs (no AE group). The distribution of age, gender and cancer type among groups is shown in **Table 2**. The distribution of AE type and grade among groups is shown in **Table 3**. Among 90 irAEs, 20 patients had grade 1, 41 had grade 2, 23 had grade 3, and 6 had grade 4 irAEs at AE onset. Two patients progressed from grade 1 to grade 2; 72 patients with grade 2, 3, or 4 irAEs received immunosuppressive therapy, including 62 completely recovered patients, 6 stable patients, and 4 patients who died.

**Table 1:** Cancer treatment methods and AE types in patients

Parameter	Name of AE	No. of patients	Group name
Immunotherapy or combination with chemotherapy	irAE	90	irAE group
	Non-irAE	70	Non-irAE group
	No irAE and no non-irAE	67	No irAE-non-irAE group
	Unclassified AE	4	–
Chemotherapy	AE	60	AE group
	No AE	27	No AE group
Total patients	–	318	–

**Table 2:** The distribution of age, gender, and cancer type among groups

Parameters	irAE group	Non-irAE group	No irAE-non-irAE group	AE group	No AE group
Gender, *n* (%)					
Male	56 (62.2)	41 (58.6)	40 (59.7)	35 (58.3)	15 (55.6)
Female	34 (37.8)	29 (41.4)	27 (40.3)	25 (41.7)	12 (44.4)
Age, years					
Median (range)	59 (35–75)	58 (36–73)	56 (33–72)	57 (34–76)	58 (32–74)
Cancer types, *n* (%)					
Lung cancer	47 (52.2)	37 (52.9)	32 (47.8)	27 (45.0)	13 (48.1)
Melanoma	10 (11.1)	8 (11.4)	13 (19.3)	0 (0)	0 (0)
Renal cell carcinoma	7 (7.8)	5 (7.1)	5 (7.5)	0 (0)	0 (0)
Cervical cancer	7 (7.8)	5 (7.1)	7 (10.4)	4 (6.7)	5 (18.5)
Thymic carcinoma	6 (6.7)	0 (0)	0 (0)	0 (0)	1 (3.7)
Bladder cancer	5 (5.6)	3 (4.3)	3 (4.5)	2 (3.3)	1 (3.7)
Hepatobiliary cancer	3 (3.3)	3 (4.3)	2 (3.0)	2 (3.3)	1 (3.7)
Gastric carcinoma	2 (2.2)	4 (5.7)	2 (3.0)	1 (1.7)	2 (7.4)
Esophageal cancer	2 (2.2)	2 (2.9)	2 (3.0)	1 (1.7)	0 (0)
Nasopharyngeal cancer	1 (1.1)	1 (1.4)	1 (1.5)	1 (1.7)	0 (0)
Lymphoma	0 (0)	2 (2.9)	0 (0)	3 (5.0)	1 (3.7)
Acute leukemia	0 (0)	0 (0)	0 (0)	19 (31.6)	3 (11.2)

**Table 3:** Distribution of AE type and grade among groups

Adverse events	irAE group					Non-irAE group					AE group				
	N	G 1	G 2	G3	G4	N	G 1	G 2	G3	G4	N	G 1	G 2	G3	G4
Hepatitis	18	0	8	8	2	13	3	1	2	7	10	0	3	4	3
Pneumonia	16	0	9	6	1	16	0	3	10	3	11	1	1	7	2
Hypothyroidism	15	7	6	1	1	1	0	0	1	0	1	0	1	0	0
Rash	14	4	7	1	2	6	0	5	1	0	7	1	4	2	0
Fever	13	5	6	2	0	4	0	1	3	0	2	0	0	2	0
Myocarditis	5	2	2	0	1	0	0	0	0	0	0	0	0	0	0
Fatigue	4	1	3	0	0	2	0	1	1	0	1	0	1	0	0
Myelosuppression	3	0	0	2	1	16	0	4	9	3	14	0	7	2	5
Cortisol reduction	3	1	0	2	0	0	0	0	0	0	1	0	1	0	0
Enteritis	2	0	1	1	0	2	0	0	2	0	6	0	2	2	2
Nephritis	2	0	0	2	0	3	1	1	0	1	0	0	0	0	0
Hyperthyroidism	1	0	1	0	0	0	0	0	0	0	0	0	0	0	0
Myositis	1	0	1	0	0	0	0	0	0	0	0	0	0	0	0
Nausea/vomiting	0	0	0	0	0	7	1	4	2	0	7	0	3	2	2
Total (No.)	97	20	44	25	8	70	5	20	31	14	60	2	23	21	14

### Diagnosis

**Figure 1:**
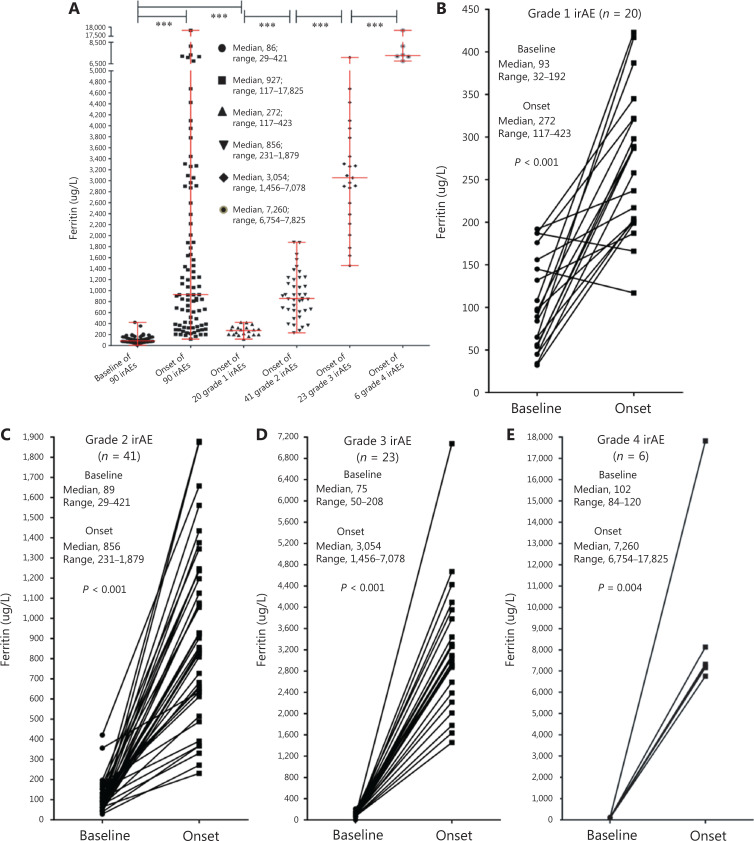
Ferritin values at baseline and AE onset in the irAE group. The bars represent the median and range. (A) Ferritin values at baseline and AE onset for 90 irAEs; ferritin at AE onset for 20 grade 1 irAEs, 41 grade 2 irAEs, 23 grade 3 irAEs, and 6 grade 4 irAEs; (B) ferritin for 20 grade 1 irAEs; (C) ferritin for 41 grade 2 irAEs; (D) ferritin for 23 grade 3 irAEs; (E) ferritin for 6 grade 4 irAEs. ***, *P* < 0.001.

At the onset of 90 irAEs, the ferritin (normal range, 35–150 µg/L) rose to a median of 927 µg/L (range, 117–17,825 µg/L) from 86 µg/L at baseline (range, 29–421 µg/L) (*P* < 0.001). The ferritin at AE onset in 20 grade 1 irAEs (median, 272 µg/L; range, 117–423 µg/L) was significantly higher than that at baseline in 90 irAEs (*P* < 0.001). The ferritin at AE onset in 41 grade 2 irAEs (median, 856 µg/L; range, 231–1,879 µg/L) was significantly higher than that in grade 1 irAEs (*P* < 0.001). In addition, we compared 23 grade 3 irAEs (median, 3,054 µg/L; range, 1,456–7,078 µg/L) with grade 2 irAEs (*P* < 0.001), and compared 6 grade 4 irAEs (median, 7,260 µg/L; range, 6,754–17,825 µg/L) with grade 3 irAEs (*P* < 0.001) (**Figure 1A**). In grade 1 irAEs, the ferritin levels at AE onset were significantly higher than those at baseline (median, 93 µg/L; range, 32–192 µg/L) (*P* < 0.001) (**Figure 1B**), and in grade 2 irAEs (**Figure 1C**), grade 3 irAEs (**Figure 1D**), and grade 4 irAEs (**Figure 1E**).

### Differential diagnosis

**Figure 2:**
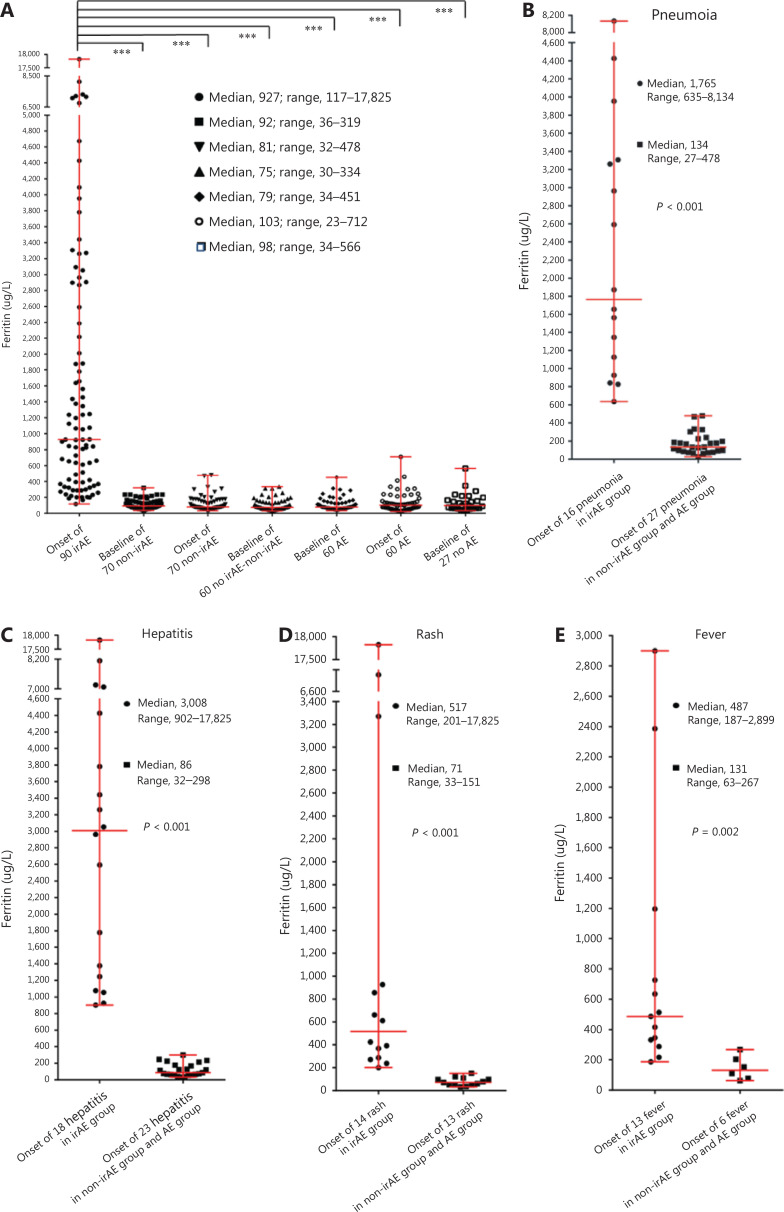
Ferritin values at baseline and after AE onset among groups. The bars represent the median and range. (A) Ferritin at AE onset in the irAE group; ferritin at baseline and AE onset in the non-irAE group and the AE group; and baseline ferritin in the no irAE-nonir AE group and no AE group; (B) ferritin at AE onset for pneumonia; (C) ferritin at AE onset for hepatitis; (D) ferritin at AE onset for rash; (E) ferritin at AE onset for fever. ***, *P* < 0.001.

The ferritin levels at AE onset in the irAE group were significantly higher than those in the non-irAE group (median, 81 µg/L; range, 32–478 µg/L) (*P* < 0.001) and the AE group (median, 103 µg/L; range, 23–712 µg/L) (*P* < 0.001) (**Figure 2A**). The ferritin at AE onset in 16 patients with pneumonia in the irAE group (median, 1,765 µg/L; range, 635–8,134 µg/L) was significantly higher than that of 27 patients with pneumonia in the non-irAE group and AE group (median, 134 µg/L; range, 27–478 µg/L) (*P* < 0.001) (**Figure 2B**). In addition, we observed 18 hepatitis cases in the irAE group (median, 3,008 µg/L; range, 902–17,825 µg/L), compared with 23 in the non-irAE group and AE group (median, 86 µg/L; range, 32–298 µg/L) (*P* < 0.001) (**Figure 2C**); 14 rash cases in the irAE group (median, 517 µg/L; range, 201–17,825 µg/L) compared with 13 in the non-irAE group and AE group (median, 71 µg/L; range, 33–151 µg/L) (*P* < 0.001) (**Figure 2D**); and 13 fever cases in the irAE group (median, 487 µg/L; range, 187–2,899 µg/L) compared with 6 in the non-irAE group and AE group (median, 131 µg/L; range, 63–267 µg/L) (*P* = 0.002) (**Figure 2E**).

### Prognosis

**Figure 3:**
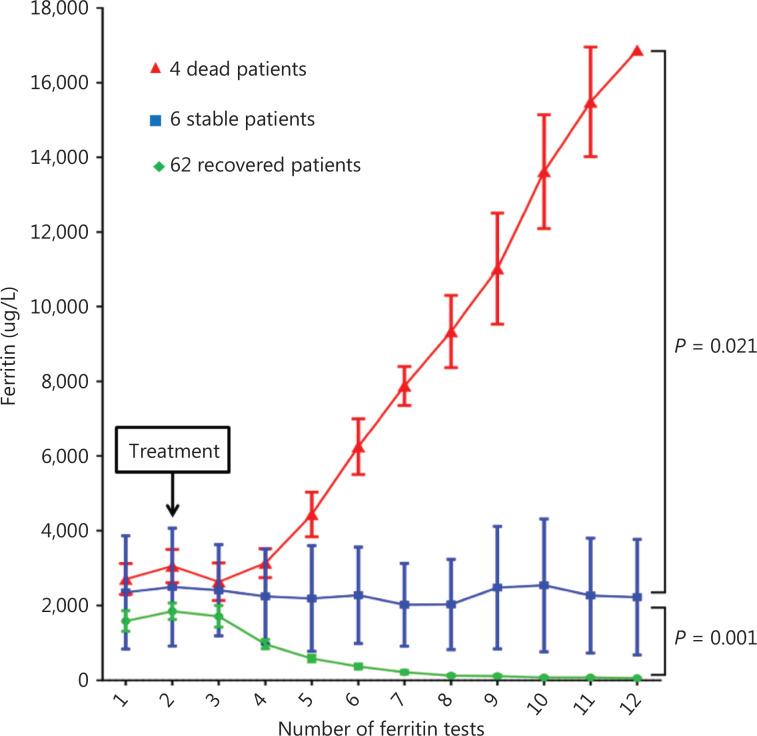
Changes in ferritin levels after treatment in 62 recovered patients, 6 stable patients, and 4 patients who died.

After treatment in the irAE group, the ferritin continuously decreased to a normal range in 62 effective patients (endpoint median, 75 µg/L; range, 34–95 µg/L); no significant changes in ferritin were observed in 4 stable patients (endpoint median, 2,317 µg/L; range, 678–4,770 µg/L); and the ferritin continued to rise in 4 patients who died (endpoint median, 16,954 µg/L; range, 11,217–17,825 µg/L) (**Figure 3**).

## Discussion

In this study, patients who received ICI therapy, chemotherapy, or ICI therapy plus chemotherapy were included. We analyzed the ferritin levels in patients with irAEs, non-irAEs, AEs caused by chemotherapy, or no AEs to better evaluate the diagnostic, differential diagnostic, and prognostic value of ferritin for irAEs induced by ICIs. The median ferritin at AE onset was significantly higher than the baseline ferritin in patients with irAEs. The median ferritin at AE onset increased with irAE grade, and statistically significant differences were observed among grades. The median ferritin at AE onset for each grade was significantly higher than the baseline ferritin for the corresponding irAE grade. Therefore, ferritin can serve as a predictive and diagnostic marker of irAEs. The median ferritin level at AE onset in patients with irAEs was significantly higher than that in patients with non-irAEs or AEs caused by chemotherapy. The ferritin level at AE onset for the irAEs pneumonia, hepatitis, rash, or fever were significantly higher than those for non-irAEs or AEs caused by chemotherapy. These results showed that the ferritin can serve as a differential diagnostic marker to distinguish irAEs from non-irAEs. After treatment in the irAE group, the ferritin continuously decreased to normal ranges in effective patients, showed no significant changes in stable patients, and continued to rise in patients who died. The change in ferritin levels reflects the therapeutic effects of ICIs on irAEs, thus indicating that ferritin is a prognostic biomarker of irAEs.

The French scientist Laufberger discovered ferritin in 1937^11^. In 1972, Addison et al.^12^ used immunoradiometric assays and validated the ability to detect ferritin in human serum. In addition, ferritin is secreted by Kupffer cells, hepatocytes, and macrophages^13^. Although numerous facets of the basic biology of serum ferritin remain unclear, various effects are increasingly being ascribed to extracellular ferritin, including newly discovered effects such as angiogenesis, immunity, cancer, inflammation, iron delivery, and signaling^14^. According to early *in vitro* studies, ferritin regulates the body’s immune function by inhibiting the function of lymphocytes^15^. Lymphocytes are activated by phytohemagglutinin and concanavalin A when human lymphocytes are treated with spleen ferritin^16^. Subsequently, another *in vivo* study has shown that ferritin suppresses immunity. Excess iron restrains the function of helper T (CD4) cells, as well as the tumoricidal action of monocytes and macrophages, and influences the distribution of the T-lymphocyte subset^17,18^. In patients with hereditary hemochromatosis, iron overload decreases the numbers and activity of CD4 cells, and increases the numbers and activity of suppressor T (CD8) cells, thus increasing the CD8:CD4 ratios^19^. Therefore, excessive iron is thought to damage the monitoring of cancer cells through these mechanisms. Serum ferritin is widely accepted to be a reactant in the acute phase and a marker of acute and chronic inflammation, and it is known to nonspecifically increase in a variety of inflammatory conditions, including chronic kidney disease, rheumatoid arthritis, and acute infection^20,21^. The elevated ferritin in these states reflects an increase in total iron storage throughout the body, but paradoxically, these stores are isolated and cannot be used for hematopoiesis, a process that leads to inflammatory anemia^22^. The relative lack of iron in inflammation and malignant tumors is speculated to be a defense mechanism that limits the use of serum iron by pathogens and tumors^23^. Serum ferritin is elevated in many malignancies^18,24^. In neuroblastoma, increased serum ferritin is directly associated with the secretion of ferritin through the tumor^25^. In our study, the ferritin levels in irAEs induced by ICIs were significantly higher than those in non-irAEs or AEs caused by chemotherapy. The mechanism underlying this phenomenon is unclear and requires further research. Studies on the mechanisms of recently emerging viruses—for instance, severe acute respiratory syndrome corona virus, Middle Eastern respiratory syndrome corona virus, and H7N9, which drive hyper cytokinemia and aggressive inflammation that lead to fatal acute lung injury—have shown that activation of the proinflammatory monocyte/macrophage system plays a crucial role in the development of diseases^26^. The occurrence and development of GVHD are always accompanied by the activation of the monocyte/macrophage system^27,28^. Whether the monocyte/macrophage system can be activated and secrete large amounts of ferritin, thus increasing serum ferritin levels in irAEs induced by ICIs, as in GVHD and severe viral infection, remains to be determined. These hypotheses must be verified and confirmed through *in vitro* and *in vivo* experiments.

## Conclusions

In conclusion, our data showed that ferritin can be used as a diagnostic, differential diagnostic, and prognostic marker for irAEs in patients with ICI treatment. The mechanism responsible for higher ferritin levels in irAEs induced by ICI than those in non-irAEs or AEs caused by chemotherapy or other therapy must be further studied.
